# P-1210. Real-World Efficacy and Safety of Meropenem-Vaborbactam in Patients with Moderate to Severe Renal Impairment

**DOI:** 10.1093/ofid/ofaf695.1403

**Published:** 2026-01-11

**Authors:** Sean R Van Helden, Kaylee Caniff, Mohammed Al Musawa, Sara Alosaimy, Abdalhamid M Lagnf, Chloe Judd, Marco R Scipione, Jing Zhao, Taryn A Eubank, Kevin W Garey, Wesley D Kufel, Tamara Krekel, Karen K Tan, Elisabeth Chandler, Jacqueline A Aramburo, Dev Chatterji, Samantha D Walker, Christine Yost, Jacqueline M Muscat, Justin A Andrade, John Cerenzio, Karan Raja, Taylor Morrisette, Sarah C J Jorgensen, Ryan P Mynatt, Travis J Carlson, David Allen, Kailynn Deronde, Ana Vega, Lilian M Abbo, Venugopalan Veena, Vasilios Athans, Steve Saw, Kimberly C Claeys, Matthew Miller, Kyle Molina, Lee Amaya, Jessica Ortwine, Michael P Veve, Michael J Rybak

**Affiliations:** Wayne State University, Eugene Applebaum College of Pharmacy and Health Sciences, Detroit, Michigan; Ferris State University, Grand Rapids, Michigan; Wayne State University, Detroit, Michigan; Seres Therapeutics, Detroit, Michigan; Wayne State University, Detroit, Michigan; Wayne State University, Detroit, Michigan; Detroit Receiving Hospital, Detroit, MI; Detroit Medical Center-Harper Hospital, Detroit, Michigan; University of Houston College of Pharmacy, Houston, TX; University of Houston, Houston, Texas; Binghamton University School of Pharmacy Sciences, Binghamton, NY; Barnes-Jewish Hospital, St. Louis, MO; Loma Linda University Medical Center, Loma Linda, California; Gulf Coast Medical Center, Fort Myers, FL; Lee Health, Fort Myers, Florida; Inova Fairfax Medical Campus, Falls Church, Virginia; University of Tennessee Medical Center, Knoxville, Tennessee; Corewell Health, Royal Oak, Michigan; Corewell Health William Beaumont University Hospital, Royal Oak, Michigan; The Brooklyn Hospital Center, Brooklyn, New York; Brooklyn Hospital, Brooklyn, New York; Clara Maass Medical Center, Belleville, NJ; Medical University of South Carolina College of Pharmacy, Charleston, South Carolina; Marshfield Clinic, Marshfield, Wisconsin; University of Kentucky, Lexington, KY; The University of Texas at Austin College of Pharmacy, San Antonio, Texas; Inova Fairfax Medical Campus, Falls Church, Virginia; Jackson Memorial Hospital, Miami, Florida; Jackson Memorial Hospital, Miami, Florida; University of Miami School of Medicine, Miami, Florida; University of Florida, College of Pharmacy, Gainesville, Florida; Hospital of the University of Pennsylvania, Philadelphia, Pennsylvania; Hospital of the University of Pennsylvania, Philadelphia, Pennsylvania; University of Maryland Baltimore, Baltimore, Maryland; Children's Hospital Colorado, Aurora, Colorado; Scripps Health, San Diego, California; Miami Cancer Institute Baptist Health, Miami, Florida; Parkland Health, Dallas, Texas; Eugene Applebaum College of Pharmacy and Health Sciences, Detroit, MI; Eugene Applebaum College of Pharmacy and Health Sciences, Detroit, Michigan

## Abstract

**Background:**

Meropenem-vaborbactam (MEV) is a novel β-lactam β-lactamase inhibitor combination approved in the United States for the treatment of complicated urinary tract infections caused by resistant organisms. Its spectrum includes carbapenem-resistant Enterobacterales and *Pseudomonas aeruginosa*. Limited data exist on the use of MEV in patients with reduced renal function. This study compared clinical characteristics and outcomes between patients with moderate to severe renal impairment and those with mild or no impairment.

**Table 1.**
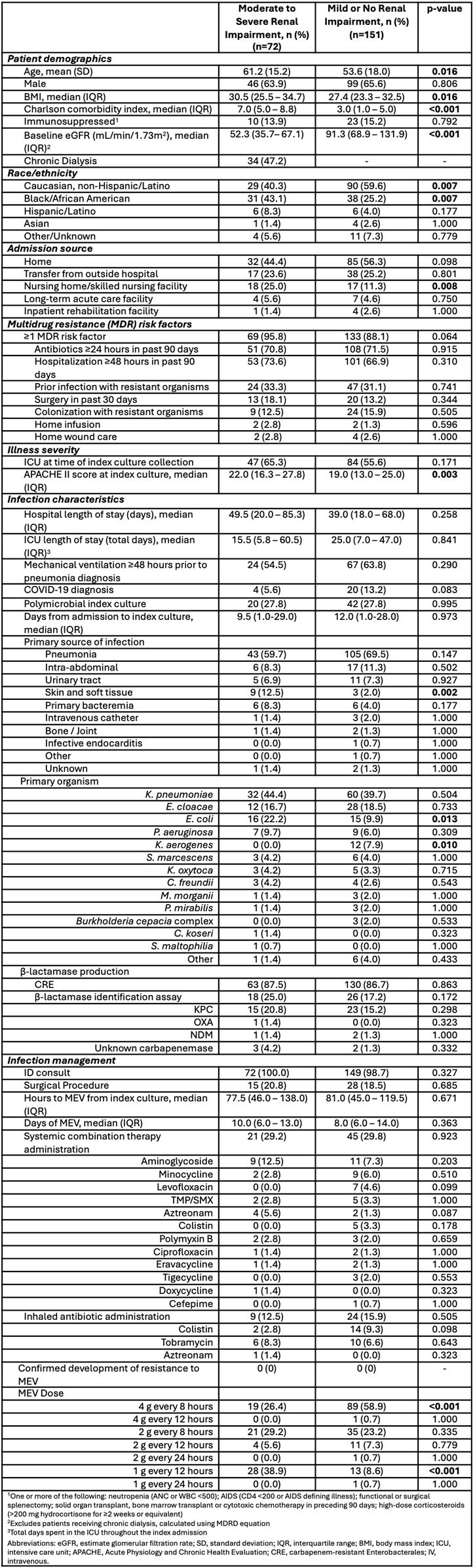
Baseline, infection, and treatment characteristics.

**Table 2.**
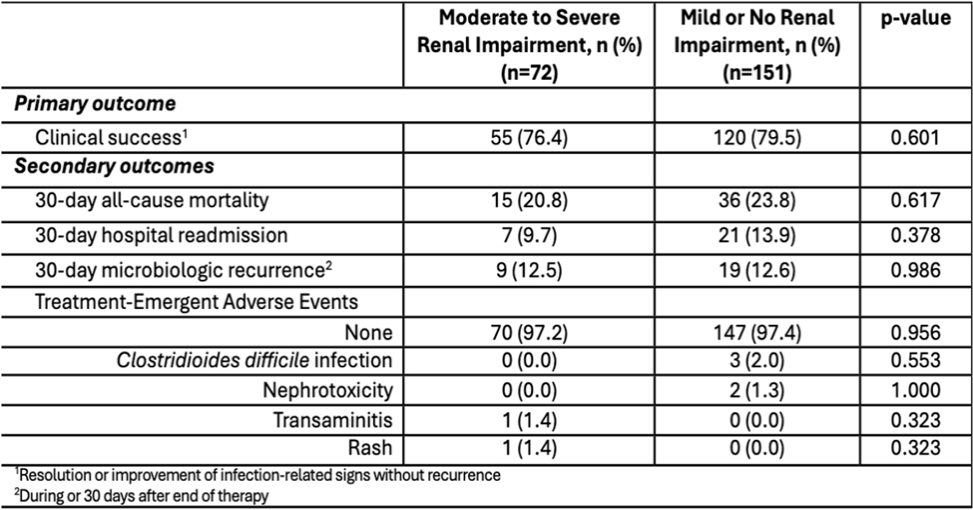
Clinical outcomes.

**Methods:**

This was a real-world, multicenter, retrospective cohort study conducted between 2017 and 2025 in adult patients who received MEV for ≥72 hours. Patients with KDOQI CKD stages 3-5 or GFR < 60 mL/min/1.73m^2^ or on chronic dialysis were assigned to the renal impairment (RI) group. All other patients were assigned to the non-impaired (NI) group. The primary outcome was clinical success, defined as resolution or improvement in signs of infection without recurrence. Secondary outcomes included 30-day all-cause mortality, 30-day microbiologic recurrence, 30-day hospital readmission, and occurrence of treatment-emergent adverse events.

**Results:**

Seventy-two patients were included in the RI group and 151 patients were included in the NI group. The median baseline eGFR was 52.3 vs. 91.3 mL/min/1.73m^2^ in the RI and NI groups, respectively. Nearly half (47%) of patients in the RI group were receiving chronic dialysis. Clinical success was achieved in 76% of patients in the RI group compared to 79% in the NI group (p=0.60). Thirty-day all-cause mortality was 20.8% in the RI group vs. 23.8% the in the NI group (p=0.62). Thirty-day microbiological recurrence and hospital readmission rates were similar between the two groups. Adverse events were rare in both groups and similar in incidence (2.8% vs. 2.6% in the RI and NI groups, respectively [p=0.96]).

**Conclusion:**

This study demonstrated the clinical outcomes of MEV when used in patients with moderate to severe renal impairment. Prospective randomized trials in this patient population are needed to validate these findings.

**Disclosures:**

Kevin W. Garey, PharmD, MS, FIDSA, FASHP, Acurx: Grant/Research Support|Merck & Co.: Grant/Research Support|Paratek Pharmaceuticals: Grant/Research Support Wesley D. Kufel, Pharm.D., BCPS, BCIDP, Merck & Co.: Grant/Research Support|Shionogi, Inc: Grant/Research Support|Shionogi, Inc: Honoraria Tamara Krekel, PharmD, BCPS, BCIDP, AbbVie: Advisor/Consultant|AbbVie: Honoraria|Shionogi: Advisor/Consultant|Shionogi: Honoraria Taylor Morrisette, PharmD, MPH, AbbVie Inc: Advisor/Consultant|AbbVie Inc.: Grant/Research Support|Copeland, Stair Valz & Lovell: Expert Testimony|Infectious Diseases Special Edition: Honoraria|Stellus Rx: Grant/Research Support Travis J. Carlson, PharmD, BCIDP, Aimmune Therapeutics, Inc.: Speaker bureau Venugopalan Veena, PharmD, Merck: Grant/Research Support Vasilios Athans, PharmD, BCIDP, Astellas Pharma: Advisor/Consultant Kimberly C. Claeys, PharmD, PhD, bioMérieux: Advisor/Consultant|bioMérieux: Honoraria Michael J. Rybak, PharmD, PhD, MPH, Abbvie: Grant/Research Support|Innoviva: Grant/Research Support|Melina: Grant/Research Support|Merck: Grant/Research Support|Shionogi: Grant/Research Support

